# Understanding the Role of Antimicrobial Peptides in Neutrophil Extracellular Traps Promoting Autoimmune Disorders

**DOI:** 10.3390/life13061307

**Published:** 2023-06-01

**Authors:** Soma Biswas, Suma Sarojini, Saranya Jayaram, Indhu Philip, Mridul Umesh, Roseanne Mascarenhas, Manikantan Pappuswamy, Balamuralikrishnan Balasubramanian, Selvaraj Arokiyaraj

**Affiliations:** 1 Department of Life Sciences, CHRIST (Deemed to be University), Bengaluru 560029, Indiamridul.umesh@christuniversity.in (M.U.);; 2 Department of Food Science and Biotechnology, College of Life Science, Sejong University, Seoul 05006, Republic of Korea

**Keywords:** antimicrobial peptides, neutrophil, autoimmune disorders, defensins, cathelicidins, cytokines

## Abstract

AMPs are small oligopeptides acting as integral elements of the innate immune system and are of tremendous potential in the medical field owing to their antimicrobial and immunomodulatory activities. They offer a multitude of immunomodulatory properties such as immune cell differentiation, inflammatory responses, cytokine production, and chemoattraction. Aberrancy in neutrophil or epithelial cell-producing AMPs leads to inflammation culminating in various autoimmune responses. In this review, we have tried to explore the role of prominent mammalian AMPs—defensins and cathelicidins, as immune regulators with special emphasis on their role in neutrophil extracellular traps which promotes autoimmune disorders. When complexed with self-DNA or self-RNA, AMPs act as autoantigens which activate plasmacytoid dendritic cells and myeloid dendritic cells leading to the production of interferons and cytokines. These trigger a series of self-directed inflammatory reactions, leading to the emergence of diverse autoimmune disorders. Since AMPs show both anti- and pro-inflammatory abilities in different ADs, there is a dire need for a complete understanding of their role before developing AMP-based therapy for autoimmune disorders.

## 1. Introduction

Initially, AMPs were thought to be substances found in tissues and secretions capable of dissolving bacteria and were termed lysozymes [1]. Lysozymes are sensitive to traces of alkalis or acids. The epidermal structures lining the respiratory tract and connective tissues have ample amounts of lysozymes. Tears, saliva, and sputum contain lysozyme for mechanically washing off the microbes as part of innate immunity. Later, it was found that lysozymes contain small peptides with antimicrobial activity, which were termed AMPs [2,3]. Today a multitude of AMPs have been discovered from diverse life forms.

AMPs are oligopeptides with host defense mechanisms found in all life forms ranging from bacteria to plants and vertebrates to invertebrates. They are usually short in length with a maximum of 60 amino acids, have a positive charge, are amphipathic in nature with hydrophobic and hydrophilic amino acids clustered together, and have a broad spectrum of antimicrobial activity [4]. Bacteriocin AMPs from bacteria are categorized into lantibiotics and non-lantibiotics. The former contains non-natural amino acids lanthionine. In 1947, Nisin, the first AMP, which is a lantibiotic, was isolated from *Lactobacillus lactis*. Mersacidin is a bacteriocin effective against antibiotic-resistant gram-positive bacteria. Since many bacteria are developing multi-drug resistance, the use of AMPs would be very crucial in pathogen control [5]. Plants and invertebrates do not have adaptive immunity; therefore, as part of innate immunity AMPs protect them from harmful microbes. In invertebrates, it has been seen that AMPs are expressed constitutively [6]. Tachyplesin and polyphemus (β-sheet AMPs) isolated from horseshoe crabs have shown antibacterial and antifungal activity. Vertebrate animals possess both inborn and acquired defenses against pathogens. AMPs in vertebrates are primarily located in epithelial cells lining the lungs, skin, mouth, etc., and in macrophages, NK (natural killer) cells, neutrophils, etc. Various AMPs have been identified and described by researchers in vertebrates such as fish, amphibians, and mammals [5,7]. Two important AMPs from vertebrates are defensins and cathelicidins, which also possess immunomodulatory functions [8]. β-defensins found in viral defense cells play a role in innate defense mechanisms against viruses [9]. AMPs that do not disrupt cell membranes have also been identified, which work intracellularly like the peptides found in neutrophil granules. Some bacteria such as *Porphyromonas gingivalis* release proteases such as gingipains (Arginine and Lysine-rich proteases) that degrade AMPs. Proteases from *Staphylococcus aureus* digest the AMPs LL-37 [10,11]. The antimicrobial abilities attributed to specific peptides within cells are a fundamental aspect of the evolutionarily preserved function of the innate immune system [12]. AMPs not only function against microbes but also have an essential role in immune regulation and controlling inflammation [8].

Neutrophils are the most important phagocytes that play an important role in managing immune defense. Neutrophil extracellular traps or NETs protect the human body against various infections and also have a role in the pathogenesis of various autoimmune disorders (ADs). The NET formation is triggered by various innate immune mediators such as reactive oxygen species (ROS), NADPH oxidase, myeloperoxidase, and neutrophil elastase and is regulated by various cytokines [13]. AMPs are significant immune system regulators that may play a role in the onset of diverse ADs. The abnormal generation of AMPs by neutrophils stimulates inflammation and leads to ADs. Dysregulation of AMPs signaling pathways acts as a triggering factor for various ADs. NETs produced by neutrophils in tissues consist of self-nucleic acids and AMPs which act for the clearance of pathogens. Excessive production of NETs under sterile conditions can activate pDCs via Toll-like receptors such as TLR9 and TLR7 triggering the production of type I interferons which act as a major initiator for ADs [14]. Apart from the role of instigating ADs, AMPs have a far more significant role in protecting cells by way of their broad-spectrum antibacterial, antiviral, antifungal, antiparasitic, anticancer, and wound-healing properties. The utility of AMPs as natural antibiotics can be better explored if the dynamics of aberrant AMPs in ADs are well understood. In this present work, we have attempted to investigate the possible role of AMPs in instigating various autoimmune disorders by the release of NETs and their potential role in developing new-generation medicines.

## 2. Neutrophil Extracellular Traps (NETs) Formation

Human physiology has always been a complex mystery by virtue of the regular upkeep of thousands of interconnected biochemical pathways, cell cycle, tissue repair and regeneration, wound healing, etc. The human immune system is an exemplary mechanism that fights against microbes with the help of memory T-cells and protective immunity. Neutrophils are a group of cells that play a vital role in immune defense, especially by being the first line of defense against infections. They were conventionally known to kill pathogens by phagocytosis and secretory antimicrobials, but later a novel function of neutrophils was identified, i.e., the formation of NETs that neutralize extracellular pathogens with the least host cell damage. NETs primarily comprise networks of extracellular fibers containing neutrophil DNA, granular and nuclear antimicrobial proteins [15], and mitochondrial DNA [16]. Detailed scanning electron microscopy (SEM) studies have revealed that NETs are composed of segments of DNA intertwined with globular protein regions measuring approximately 15–17 nm and 25 nm in diameter, respectively. These components further aggregate to form larger threads with a diameter of 50 nm [15]. The antimicrobial protein composition of NETs has been elucidated further through immunofluorescence analyses that exhibited the presence of proteins such as gelatinase (from tertiary granules), lactoferrin (from specific granules), neutrophil elastase, cathepsin G, myeloperoxidase (from azurophilic granules) and other cytoplasmic proteins. Additionally, neutrophil-stimulated NETs contain proteins such as histones, calprotectin, cathelicidins, actin, and defensins. Among these, phorbol-12-myristate-13-acetate (PMA) stimulates protein kinase C (PKC). This further initiates the release of reactive oxygen species (ROS) [17]. Recent studies hypothesize that based on the stimuli, the composition of antimicrobial proteins in NETs varies drastically. This rich arsenal of AMPs is stored in specialized granules and their release is tightly regulated in lieu of protecting host cells from damage. Deployment of these neutrophil-associated antimicrobial proteins is regulated through degranulation, phagocytosis, and release of NETs.

**Figure 1:**
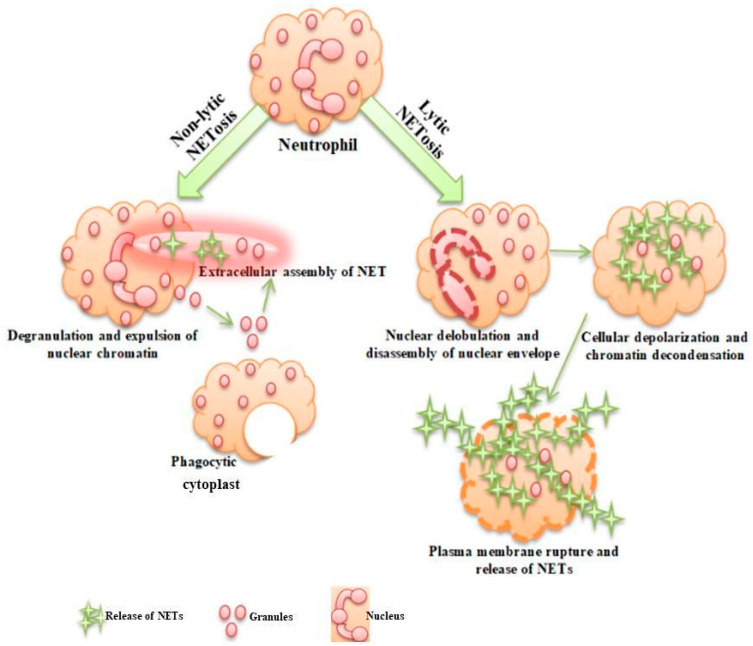
Representation of neutrophil extracellular trap formation through lytic and non-lytic NETosis pathways.

The release of NETs occurs primarily through NETosis brought about by cell lysis. As depicted in Figure 1, neutrophils initiate this process by depolarizing and arresting their actin dynamics [18]. Following this neutrophil activation, their nuclear envelope undergoes disassembly and nuclear chromatin undergoes decondensation into cellular cytoplasm thus mixing with cytoplasmic and granule components [19]. Accompanied by plasma membrane permeabilization, NETs begin to expand into extracellular space around 4–8 h after this neutrophil activation. Alternatively, NETosis that is not regulated by cell death occurs when chromatin and granular components of NETs are rapidly released within minutes of encountering *Staphylococcus aureus* [20]. Here, anucleated cytoplasts and neutrophils that first arrive at sites of infection mount this rapid non-lytic NETosis response. During infections, NETs persist for several days. The clearance of NETs has been poorly understood; they are believed to be broken down by DNase I, a secreted protein found in plasma. However, NET proteins were found to persist even long after DNA degradation [21], suggesting the involvement of additional mechanisms for clearance. NETs are considered to be large extracellular web-like structures made up of granular and cytosolic proteins (AMPs) embedded in decondensed neutrophil chromatin. Thus, during pathogenesis, NETs help in trapping and exterminating extracellular pathogens while also preventing the dissemination of fungi and bacteria. On the downside, the pathogenesis of immune-related disorders is greatly contributed by dysregulated NETs [22].

## 3. Mammalian AMPs

The AMPs are present in many parts of the human body such as skin, mucosae, etc., that are exposed to microbes. AMPs are typically produced together as a mixture of several peptides, with tissue-specific unique AMP combinations. While specific AMPs are more prevalent in particular parts of the body, very few are exclusively produced by a single tissue or cell type. Almost all AMPs have multiple functions. AMPs, such as defensins and cathelicidins, were initially identified and studied due to their antimicrobial properties. Defensins and cathelicidins possess various immunomodulatory activities apart from their broad spectrum of activity against pathogens.

### 3.1. Defensins

Defensins have a β-sheet structure with 6 Cysteine residues forming 3 disulfide bonds. Based on the arrangement of disulfide bonds, they are subdivided into β-defensins, α-defensins, and θ-defensins. Defensins kill both gram-positive as well as gram-negative bacteria, viruses, protozoa, and fungi. β-defensins interact with lipid II on the bacterial membrane and disrupt it. Defensins also act as immunomodulators by inducing the generation of pro-inflammatory cytokines, which function as chemokines for neutrophils and facilitate macrophage phagocytosis [23]. They act as chemoattractants for immune cells. Studies suggest that the reduction of a disulfide bond in β-defensin has resulted in increased antimicrobial activity. Thus, disulfide bonds may act as regulators for antimicrobial activity [24].

α-defensin in humans is encoded by chromosomal locus 8p23.1. Although AMPs are encoded by 3 exons, usually the third exon codes for mature AMP. α-defensins, HNP1-4 (human neutrophil peptide) are found in high concentration in neutrophil granules and are released upon degranulation of the neutrophil. α-defensins are also found to degranulate mast cells, inhibit glucocorticosteroid production, act as chemokines for monocytes, and are mitogenic for epithelial cells. Activation of human β-defensins HBD2 and HBD3 occurs in response to factors such as tissue injury, bacterial components such as lipopolysaccharide, lipoteichoic acid, as well as pro-inflammatory cytokines TNF-α and Interleukin-1α [23,25]. The Paneth cells of the small intestine also contain α-defensins. The inactive defensins consist of a signal peptide of 19 amino acids, a cationic mature defensin peptide of 29–40 amino acids, and an anionic pro-peptide of 45 amino acids. The cationic α-defensins get activated by the removal of the anionic propeptide. Defensins are known to inhibit PKC Phospholipid/Ca^2+^ protein kinase [26]. A new AMP bioinspired peptide CaDef2.1G27-K44 from *Capsicum annuum* L. has been found to exhibit increased anticandidal and antimycobacterial activity with low hemolytic and cytotoxic properties against mammalian cells [27].

### 3.2. Cathelicidins

They are transcribed from pre-proteins containing 4 exons. Signal peptide 29–30 amino acids long is encoded by the first exon; the Cathelin domain is encoded by the next two exons and the mature peptide is encoded by exon 4. The antimicrobial domain is 12–100 amino acids long. The gene encoding cathelicidin is found in many mammals, fishes, birds, lizards, etc. The sequence of the N-terminal cathelin domain has been highly conserved and has protease inhibitor activity along with anionic antimicrobial activity against bacteria such as *S. aureus* and *E. coli*. The sequence of C-terminals of AMPs is highly divergent with differences in size and positions of folds. The predominant form of cathelicidin is a linear peptide that adopts an α-helical conformation upon interacting with a lipid membrane [28,29]. LL-37/human cationic antimicrobial protein (hCAP18) mainly expressed by epithelial cells and neutrophils is the C-terminal part of the only identified human cathelicidin. Granules of neutrophils contain high concentrations of cathelicidin. They are also expressed in epithelial cells, mucosal cells of the intestines, genital, respiratory, and urinary tract, and also in monocytes, mast cells, B-cells, and NK cells [30,31]. The mature cathelicidin may have α or β helices or polyproline helix type II or sometimes an unordered structure [32]. Some of the defensin and cathelicidin peptides with their essential role in innate immunity are enlisted in Table 1.

## 4. Mechanism of Action of AMPs

The use of microscopy has helped greatly to understand the underlying mechanism of action and target sites where these peptides perform their function. Microscopic analyses reveal that peptides with distinct characteristics exhibit variances in their target locations and mechanisms of action and they work differently for different microbial cells. The makeup of amino acids, the positive charge, and the size of the peptide enable the binding of the AMP to the negatively charged bilayer of the target cell’s membrane.

### 4.1. AMPs as Antimicrobials

AMPs such as HBD2 have been found to bind to the phospholipid bilayer and induce efflux of intracellular components leading to the death of the pathogen. Neutrophils create extracellular traps containing AMPs that can destroy pathogens [44]. Apart from AMPs acting on membrane bilayers leading to microbial lysis, they are also found to have intracellular targets. Several proline-rich peptides and arginine-rich peptides translocate through the cytoplasmic membrane and act intracellularly by inhibiting protein synthesis, nucleic-acid synthesis, and enzyme synthesis. HD-5 peptide has been implicated in the successful killing of both gram-negative and gram-positive bacteria by disrupting their bilipid layer. HD-5 has the ability to enhance bacterial membrane permeability, which makes it effective against both gram-positive and gram-negative bacteria. Additionally, HD-5 has been found to obstruct cell replication by binding to DNA [38]. Different mechanisms have been proposed for the antimicrobial activity of AMPs. The cationic peptide binds to anionic phospholipids and phosphate groups on the teichoic acid in gram-positive bacteria and lipopolysaccharide in gram-negative bacteria [45]. The peptides are held parallel to the membrane surface at low peptide-to-lipid ratios and held perpendicular at high peptide-to-lipid ratios, thus forming transmembrane pores [46]. Different transmembrane pore-forming models include the toroidal pore model, barrel stave model, and carpet model. In the barrel stave model, the peptide helices create pores on the plasma membrane bilipid layer with hydrophobic amino acids facing the lipid bilayer and hydrophilic amino acids in the inner portion of the pore. In the carpet model, peptide helices line themselves parallel to the plane of the membrane bilayer, in the form of a carpet covering the bilayer. At a high peptide concentration, they tend to disrupt the bilayer and form micelles. The peptide helices interact with the lipid bilayer in a way that allows the monolayer to bend along with the pore in the toroidal model. The polar groups of the lipid bilayer interact with the polar side of the peptide helix forming a continuous toroidal hole or pore [47].

### 4.2. Role of AMPs in Physiological Conditions

When the immune system encounters microbes, AMPs are produced by various immune cells such as monocytes, NK cells, neutrophils, macrophages, etc. They are the first ones to interact with the invading microbes. AMPs at the site of encounter initiate an immune response which further recruits other immune cells and reduces inflammation. AMPs give many immune responses such as attraction, activation, and differentiation of WBCs, stimulation of angiogenesis, and reduction of inflammation [48]. Human AMPs, defensins, and LL-37 act as chemoattractants for mast cells, leukocytes, and dendritic cells. Innate defense regulators (IDR) are synthetic AMPs. IDR-1018 has been found to reduce inflammation in mice when infected with malaria, it does not have any direct antimalarial activity and reduces the chances of death by malaria [5].

α-defensins such as HNP-1 and 2 are chemotactic towards monocytes, CD4+ and CD8+ T-cells, neutrophils, and mast cells. They recruit these cells with the help of a G-protein-coupled receptor—formyl peptide receptor-like 1 (FPRL1). Similarly, HBD1 and HBD2 show chemotaxis towards memory T-cells and immature dendritic cells (iDC) via GPCR CCR6. HBD-2, 3, and 4 also attract mast cells and trigger the release of granules from mast cells, the production of prostaglandins D2, and intracellular calcium mobilization. HBD3 also interacts with GPCR CXCR4 on T-cells, which is an essential co-receptor for the binding of HIV-1. HBD3 basically inhibits the binding of HIV-1 to T-cells by internalizing the CXCR4 receptor [9,49,50]. Studies have shown that cells that over-express defensins have greater Th1 response and also help induction of IL2, IFN gamma, Nk cells, and Tc-cells (cytotoxic T-cells) in mice and, hence, offer greater protection against cancer cells [51].

In psoriasis patients, LL-37 can form complexes with extracellular self-DNA and promote inflammation by activating TLR9 response in plasmacytoid dendritic cells (pDCs). The pro-inflammatory activity of cathelicidin has been observed in diseases such as atherosclerosis, diabetes, and SLE [52]. CAMP genes are usually constitutively expressed but they can also be induced. Increased expression of genes has been observed in some diseases such as lupus, psoriasis, and cystic fibrosis. CAMP gene expression can be induced by certain compounds such as butyrate, phenylbutyrate, lithocholic acid, vitamin D, and insulin-like growth factor 1 in vitro [29]. Unlike defensins, cathelicidin expression depends more on vitamin D than on TLR or cytokines. The connection between cathelicidin and vitamin D has also been seen in carcinogenesis [53].

LL-37 inhibits LPS binding to LPS-binding protein, thereby neutralizing endotoxin, thus inhibiting LPS-induced pro-inflammatory responses. LL-37 also induces the expression of signaling proteins (chemokines) such as IL-8 and MCP-1 to recruit other immune cells. It has also been seen that AMPs with different disulfide linkage patterns recruit monocytes differently. This suggests that the pattern of peptide folding gives primary clues in recruiting cells of the innate and adaptive immune system [54]. Mast cell (MCs) granules release cathelicidin in response to infection. Surface components of many viruses can be recognized by MCs. The degranulation of MCs gets activated via membrane-activated pathways over a TLR-signalling-activated pathway. MCs in response to viral infection produce IFN and TNF-α which modulate T-cell response [55].

Findings suggest that AMPs act as strong immune adjuvants and activate T cell-mediated humoral immunity, though the proper mechanism has not yet been reported. AMPs act as immune modulators by inducing cytokine expression upon antigen encounter, inducing maturation of dendritic cells, and activation of T-cells [9]. Defensins act as immunomodulators by promoting chemotactic activity, antigen presentation, and secretion of proinflammatory cytokines. Defensins act as the first line of defense as well as connectors of the innate and adaptive immune responses. They have a role in the pathogenesis of rheumatic diseases. The chemotactic activity of defensins helps to attract macrophages, immature dendritic cells, and monocytes to the inflammation site. T cells are also attracted by defensins in order to interact with APCs [12]. Broad-spectrum antimicrobial activity against both gram-negative and gram-positive bacteria has been depicted by PNKL, identified from the small intestine. It was observed that PNKL led to a decrease in the expressions of inflammatory cytokines IL-6, NF-κB, TNF-α, Caspase-3, and Caspase-9 in the *E. coli* K88-challenged cells. These properties offer PNKL to be a substitute for some antibiotics [42]. AMP production using (clustered regularly interspaced short palindromic repeats) CRISPR technology was tried out in *Bombyx mori* cells; CRISPR/Cas9 could successfully induce site-specific mutation of the cactus gene that could produce AMPs through gene editing [56]. Thus, CRISPR can be used as a next-generation gene editing tool to increase the recombinant biosynthesis of AMPs [43].

## 5. Neutrophil Extracellular Traps Triggering Autoimmune Responses

Neutrophils play a crucial role as the frontline defenders of the immune system, serving as the primary cells in the initial defense against pathogens. NETs, consisting of extracellular fibers, DNA, histones, and cationic peptides play a very important role in innate immunity. Different mechanisms that neutrophils perform include phagocytosis, degranulation, cytokine, and NET production. ROS acts as a secondary messenger in NETosis. NETosis is triggered through TLRs and complement system C3 protein [57]. Depending on the microbe size, neutrophils decide whether to launch the release of NETs into the extracellular environment to trap the microbes. Small-sized microbes are unable to initiate NETosis as they are readily taken up by phagocytosis and fuse with neutrophil granules. Microbes that are larger in size cannot be engulfed by phagocytosis and thus the granular contents are released to form NETs. Sometimes microbes may evade phagocytosis, so microbe size cannot be a determining factor for antimicrobial response by neutrophils [58]. Mechanisms that lead to NET release include suicidal, noncanonical, and vital NETosis [59]. Most AMPs are upregulated in ADs, for example, LL-37 is upregulated and is associated with the pathogenesis of lupus, psoriasis, and RA. Studies show that gut microbiota can induce the expression of AMPs and trigger autoimmune responses. AMPs can be used as clinical or diagnostic markers for the detection of various ADs, as specific AMPs were found to be upregulated in specific ADs [14]. This review focuses on the role of AMPs in the development of different ADs. In psoriasis, self-DNA forms a complex with LL-37 and activates pDCs via TLR9 and releases IFN-α which triggers the development of psoriasis. Even LL-37 can activate T-cells as auto-antigen in psoriasis. Increased infiltration of neutrophils and NK cells produces AMPs which play an important role in the pathogenesis of psoriasis [60]. Upregulation of human α-defensin 3 has been seen in RA. Neutrophils play an important role in the pathogenesis of RA by releasing cytokines, chemokines, auto-antigens, and the formation of NETs. Gut microbiota control the expression of CRAMP which is found to be upregulated in type I diabetes. By changing the gut microbiota, AMP expression can be reduced which can further reduce the chances of autoimmune diabetes. Modulating gut microbiota would help to change the AMP expression, thereby preventing many different types of ADs [61].

The release of NET along with AMPs from neutrophils kills microbes and also contributes to the pathogenesis of various infectious and non-infectious diseases. ROS, LPS, chemokine, and CXCL8 are inducers of NETosis. Neutrophils can be detrimental to self-tissues as seen in ADs. NETosis has been reported in many species such as fishes, humans, mice, chickens, horses, etc. Histones present in NETs also have antimicrobial activity but it is not exactly clear how they work in extracellular environments [62]. The role of anti-viral factor α-defensins in NETs is not clearly understood; possibly HIV gets trapped by the web-like structure of NETs and later cleared by α-defensins and myeloperoxidase. The pathogenomics role of NETs has been observed in acute viral bronchiolitis caused by a respiratory syncytial virus. NETosis is both useful as well as harmful for the organism; useful because it helps to clear the invading pathogen and harmful as it contributes to the pathogenesis of many diseases [63]. A delicate balance is key to the well-being of the species.

The elevated levels of neutrophils are early indicators of the presence of SARS-CoV2 in persons suffering from COVID-19. The serum from COVID-19 patients shows the presence of DNA, histones (Cit-H3), and myeloperoxidase (MPO-DNA) which are specific components for NETs that release inflammatory cytokines such as IL-1β, and IL-6 [64]. Pieces of evidence indicate that the release of NETs may have contributed to the inflammatory storm that had led to respiratory failure. The amount of cell-free DNA, histones, and myeloperoxidase was found to be higher in patients using ventilators. The serum of COVID-19 patients also showed healthy neutrophils being triggered to abruptly undergo NETosis [65]. FDA-approved drug dipyridamole, an adenosine-receptor agonist, has been found to inhibit NET formation by the activation of the adenosine A2A receptor [66]. In ADs such as psoriasis, PMN releases NETs which contain self-DNA-LL-37 and self-RNA-LL-37 as immunostimulators that activate pDCs via TLRs to release inflammatory cytokine production, leading to inflammation and infiltration of immune cells such as dendritic cells, T-cells, and PMN neutrophils in psoriatic lesions. pDCs are activated by a complex of nucleic acid and antimicrobial self-peptide LL-37, which is present in high concentrations on psoriatic skin [67].

Patients with severe neutropenia when infected with bacteria or viruses release molecules from neutrophils as host defense. These patients have higher chances of developing ADs. The development of anti-proteolytic and anti-antimicrobial molecules against specific proteases and peptides found in neutrophils would be of prime importance in treating many diseases [68]. One study reported that in sepsis when LL-37 is administered, it induces neutrophils to release ectosomes with an antimicrobial activity which decreases the bacterial load in cecal ligation and puncture (CLP) mice. The study also revealed that LL-37 has the ability to block the attachment of LPS to CD14 and TLR4 receptors on the cell surface, thereby inhibiting LPS-induced cell apoptosis by gram-negative bacteria. LL-37 also inhibits apoptosis of neutrophils, chemotaxis, and intracellular Ca2+ mobilization via a formyl peptide receptor-like 1 and purinergic receptor. Findings show that FPR2 (not P2X7) and CXCR2 act as receptors for LL-37 to release ectosome from neutrophils and LL-37 acts as a protective shield in sepsis by the release of ectosomes [69].

LL-37 is abundantly found in NETs. The cationic property of LL-37 helps to protect NETs from bacterial nuclease degradation. Thus, NETs containing LL-37 exhibit higher resistance against *S. aureus* compared to NETs that do not contain LL-37. Bacteria such as *S.aureus*, *S. pneumonia*, and group A *Streptococci* (GAS) release nucleases that degrade NETs and allow the spread of infection [70]. The elevated levels of LL-37 in NETs could be an important factor responsible for the antimicrobial activity of NETs. Some studies also show that LL-37 loses its antimicrobial property once bound with DNA in NETs. Therefore, the exact function of LL-37 in NETs remains uncertain. The abundance of cathelicidin LL-37 in NETs also shows the importance of this peptide in NET formation and the maintenance of its longevity [71]. IL-17 and IL-23 are the cytokines that are targeted in psoriasis for making drugs and are found to be very effective in treating this disease. IL-17 and IL-23 are mostly produced by mast cells and neutrophils in skin cells. Increased expression of IL-17 by Th17 cells is seen in lupus, asthma, multiple sclerosis, RA, Crohn’s disease, autoimmune uveitis, and ankylosing spondylitis [72].

Histones in NETs confer antimicrobial activity at a very low concentration. NETs were found to be in high concentration near inflammatory sites. NETs not only have antimicrobial properties but also act as physical barriers for controlling the spread of microbes. The extracellular release of histones in NETs could possibly trigger the development of autoimmune activity as in lupus [15]. pDCs express TLR7 and TLR9 through which they sense viral or microbial DNA and produce interferons. Normally, pDCs do not respond to self-DNA as they have fewer CpG motifs and are usually masked by methylation. pDCs can sense self-DNA coupled with AMPs (LL-37) and trigger innate immune activation which leads to an autoimmune response. Activation of pDCs and release of IFNs have been reported in ADs [73]. Self-RNA-LL-37 also activates mDCs (myeloid dendritic cells) through TLR8 with the release of proinflammatory cytokines such as TNF-α and IL-6. Thus, mDCs undergo differentiation into mature dendritic cells. Self-DNA-LL-37 cannot activate mDCs. Studies show that self-RNA-LL-37 along with activated pDCs producing IFN-α is essential in the activation and maturation of mDCs. Excessive activation of pDCs and mDCs can result in the stimulation of self-antigen-specific T-cells at elevated levels, which will induce an autoimmune response. Strategies need to be explored to treat ADs by finding out ways to suppress the production of LL-37 or prevent the formation of the complex LL-37-nucleic acid [74]. Blocking LL-37 with a specific antibody was found to restrict the production of IFN-α. Also, IFN-α production by LL-37-self DNA has been reported to be blocked by chloroquine [75].

## 6. Role of AMPs in Autoimmune Disorders

There is growing evidence to suggest that AMPs have significant involvement in the immune response in autoimmune disease-affected individuals. The dysregulation of AMP production has been identified as a contributing factor to the pathophysiology of these disorders. Neutrophils undergo NETosis, a process in which they release their contents; these innate immune complexes are identified by autoantibodies as self-cells leading to inflammation of joints and tissues. Neutrophils are activated by pro-inflammatory cytokines and chemokines, which prompt them to migrate to the site of inflammation [73]. They then release cytokines which act as chemoattractants and recruit more neutrophils at the site of inflammation. During NETosis, chromatin in neutrophils undergoes remodeling and binds to AMPs effectively; it is released as a result of NETosis. The release of histones and DNA during NETosis acts as antigen presentation in many rheumatic diseases [76].

### 6.1. Rheumatic Arthritis (RA)

**Figure 2:**
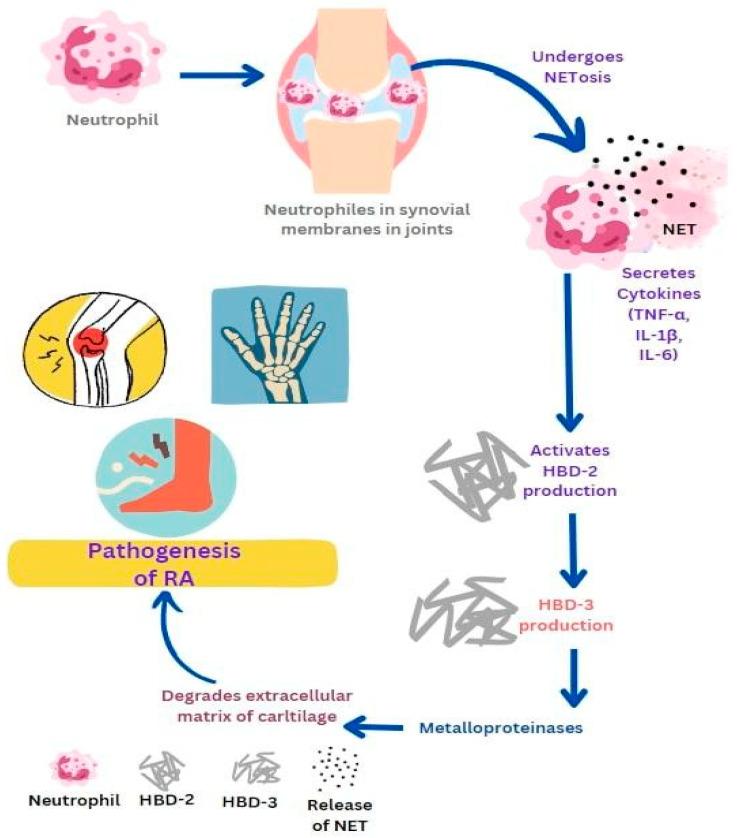
Role of antimicrobial peptides in promoting the pathogenesis of rheumatoid arthritis (RA). Neutrophil activation in joints leads to the production of cytokines and chemokines which further promote neutrophil recruitment and NETs formation. Activated NETs promote inflammation by producing cytokines such as TNF-α, IL-6, and IL-1β which then activate the production of HBD-2 and HBD-3. HBD-3 activates metalloproteinases which play an important role in cartilage destruction.

RA is a type of inflammatory condition that affects joints and causes long-term damage to cartilage and bones. It is caused due to the accumulation of autoantibodies. In RA patients, α-defensins 1–3 (HNPs) were identified in synovial fluid, which acts as biomarkers [32]. In rats with arthritis, the synovial membranes in their joints had the highest concentration of cathelicidins. Neutrophils, a type of immune cell, are pushed into the inflamed joints by granulocyte colony-stimulating factor, which also triggers their production. Leukotriene B4 and its receptor promote the recruitment of neutrophils into the joints by causing dysregulation of T-helper type 17 [77]. Neutrophil activation leads to the production of cytokine IL-1β in synovial fluid which then produces chemokines and promotes neutrophil recruitment into the joints. Rescinding of RA may be possible by reducing neutrophils moving towards the synovial fluid by making changes in neutrophil adhesion and chemotaxis in the joints. Neutrophils in the bloodstream and synovial fluid are more likely to form NETs [78]. RA NETs promote inflammation by activating cytokines, chemokines, and adhesion molecules in synovial fluids. In RA, the level of NETosis is related to anti-citrullinated peptide antibodies [79]. The proinflammatory cytokines such as TNF-α, IL-6, and IL-1β can activate the production of HBD-2 in chondrocytes. HBD3, another β-defensin, activates metalloproteinases by chondrocytes that play an important role in cartilage destruction (Figure 2). Elevated levels of the human cathelicidin LL-37 and its processing enzyme proteinase 3 were observed to be associated with this disease. LL-37 also triggers apoptosis of osteoblasts, which results in reduced bone formation in the joints affected by arthritis [32]. The pathogenesis of rheumatoid arthritis gets triggered by neutrophil-releasing substances that promote inflammation and attract immune cells. The complete etiology and pathogenesis of RA have not been completely elucidated, but the use of some anti-rheumatic medications such as adalimumab and etanercept has been shown to noticeably decrease the expression of LL-37. This suggests a connection between LL-37 expression and the severity of RA [61].

### 6.2. Systemic Lupus Erythematosus (SLE)

SLE is a multifactorial autoimmune problem that affects the entire body and occurs when the immune system loses control and attacks the body’s own tissues. It is caused by the presence of autoantibodies that target nuclear antigens. SLE is characterized by symptoms that potentially attack almost every organ system, although it primarily affects the heart, joints, skin, lungs, blood vessels, liver, kidneys, and nervous system. Increased NETosis by SLE neutrophils were observed upon stimulation by antibodies against LL-37 [57]. In SLE, neutrophils are thought to be responsible for the pathogenesis of the disease. Activated neutrophils release AMPs that complexed with self-DNA and trigger pDCs activation via TLR9. AMP-self-DNA complexes in NETs possibly act as auto-antigens against which auto-antibodies are made in SLE. A link has been proposed between the activation of neutrophils, pDCs activation, and the development of autoimmunity in SLE [78]. Activation of pDCs releases type I interferon-α which increases the monocytes’ differentiation into dendritic cells, which subsequently stimulate the activation of self-reactive B and T cells, thereby promoting autoimmunity in SLE. High numbers of apoptotic neutrophils, as well as immature neutrophils, were detected in patients’ blood. Self-DNA are very unstable in the extracellular environment and degrade easily [76], but when self-DNA are complexed in an immunogenic complex, they trigger the activation of pDCs via TLR9.

**Figure 3:**
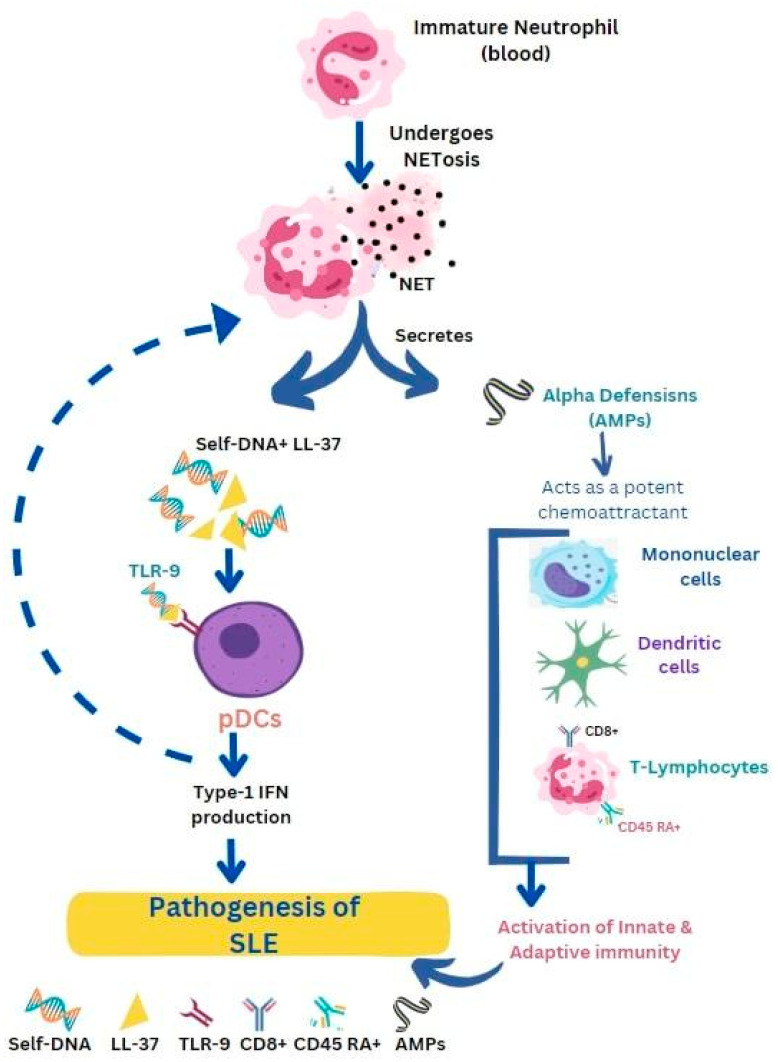
Antimicrobial peptides such as LL-37 and defensins facilitate the progression of systemic lupus erythematosus (SLE). Release of immunogenic complexes (self DNA + LL-37) from apoptotic neutrophils prompts the activation of pDCs via Toll-like receptors (TLR-9). Activated pDCs release type-1 IFN which further promotes NETosis. α-defensin secretion from activated neutrophils serves as a chemoattractant for immune cells, activating innate and adaptive immunity leading to the SLE condition.

Possibly, AMPs such as LL-37 and HNPs play an important role in making self-DNA immunogenic by inducing aggregation of self-DNA into an insoluble particle, against which auto-antibodies are released. These immunogenic complexes having self-DNA and AMPs are released from apoptotic neutrophils during NETosis (Figure 3) [32]. In SLE patients, auto-antibodies are released not only against self-DNA but also against AMPs in NETs. These auto-antibodies in the immune complex interact with FcGamma RII on pDCs and trigger receptor-mediated endocytosis of self-DNA. Neutrophils in SLE patients express high amounts of surface LL-37 and HNPs against which antibodies are secreted. Anti-LL-37 and anti-HNP trigger the release of NETs from neutrophils. Thus, circulating neutrophils in SLE release large amounts of NETs in response to circulating antibodies against AMPs [75]. In patients with SLE, neutrophil activation results in α-defensin secretion. These, in turn, function as chemoattractants for T-lymphocytes, mononuclear cells, and dendritic cells. These together activate the innate and adaptive immunity leading to the diseased state [32]. The pathogenicity of SLE stems from the involvement of cathelicidin, its presence in NETs, and its capability to create and maintain immune complexes with DNA and autoantibodies. As mentioned earlier, these complexes stimulate the secretion of type I interferon (IFN) by plasmacytoid dendritic cells (pDCs) and the production of autoantibodies by B cells [14]. Nevertheless, the precise role of each cell in SLE is not yet completely understood.

### 6.3. Type I Diabetes Mellitus (TIDM)

TIDM is a disease caused by an autoimmune response that destroys the pancreatic β-cells responsible for producing insulin. In TIDM, the T cells that are self-reactive activate the insulin-producing β-cells in the pancreas. Levels of LL-37 and β-defensins (HBD1) levels are decreased in comparison to healthy individuals. Studies show that AMP dysregulation might lead to TIDM [32]. Research has also demonstrated the crucial role of gut microbiota in the pancreas in regulating autoimmune diabetes. Gut microbiota helps to modulate CRAMP production; CRAMP regulates the pancreatic immune environment by inducing lymphoid and myeloid regulatory cells to prevent autoimmune diabetes [52,80].

Mouse studies have elucidated the involvement of gut microbiota in the development of autoimmune TIDM. Reduction of CRAMP expression caused dysbiosis and an increase in pancreatic autoimmune response, further initiating the development of diabetes. An increase in CRAMP expression by local treatment reinstated colonic homeostasis. Infections by enteric pathogens can upset gut barriers leading to the development of TIDM [81]. *Citrobacter rodentium* was used as a model organism to represent colonic infection with disruption in the gut barrier. The infection caused a reduction of CRAMP production, activation in the interferon-gamma (IFN-γ)+ cells, and an increase in T1DM. They concluded that cathelicidin supplementation can bring back healthy gut barriers and thus prevent T1DM [82]. Like cathelicidins, β defensins also show immunomodulatory properties such as chemotaxis, immune cell modulation, and regulation of both adaptive and innate immune responses. The gut microbiota has the ability to regulate the expression of CRAMP in pancreatic β-cells by producing short-chain fatty acids. Therefore, modifying the gut microbiota could be a potential approach to reduce the pathogenesis of autoimmune diabetes by altering the expression of AMPs [81]. In a study, Miani et al. [83] reported the role of mBD14 (mouse β-defensin 14) against T1DM. The AMPs stimulated the production of IL-4 by B-cells and caused the β cells of the pancreas to express transgenic IL-4. As a result, diabetes was prevented in NOD (non-obese diabetics) mice [83]. Modifying intestinal antimicrobial peptides could be seen as a significant therapeutic strategy to safeguard at-risk children from developing autoimmune diabetes.

### 6.4. Psoriasis

Psoriasis is a chronic inflammatory disease with symptoms affecting the skin with the presence of inflammatory plaques and characterized by complex immune responses wherein molecular, cellular, and environmental factors play important roles in the disease development. Injury, infection, or genetic factors trigger the keratinocytes to produce elevated concentrations of AMPs such as LL-37, which can bind to self-DNA to form an immunogenic complex that activates pDCs through TLR9. Increased TLR9 production and expression leads to increased type I IFN and TNF-α expression and inflammation. Human cathelicidin (LL-37) can form a complex with self-RNA to activate pDCs through TLR7 and mDCs through TLR8. Additionally, neutrophils are also capable of inducing the expression of human β-defensin 2 (HBD2) in keratinocytes of psoriatic skin through the formation of NETs [58,59]. LL-37 is also identified as a self-antigen for circulating T-cells in psoriasis.

**Figure 4:**
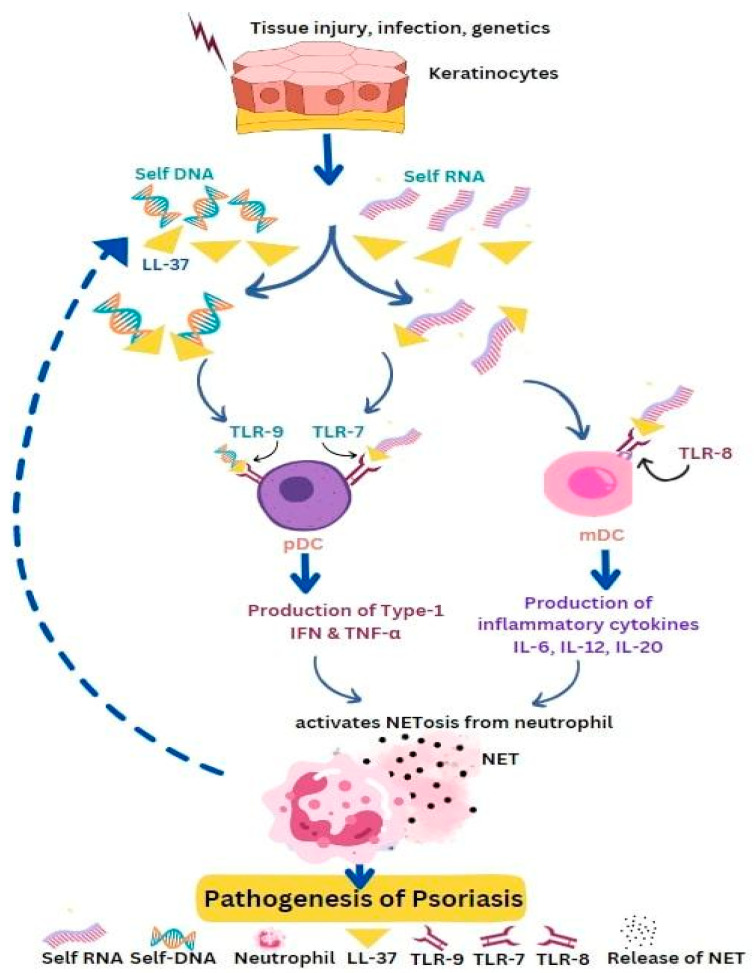
Self-DNA/LL-37 and self-RNA/LL-37 promote the pathogenesis of psoriasis. Keratinocytes get activated under the pressure of tissue injury, infection, or genetic factors and express high levels of AMPs and nucleic acids, which form immunogenic complexes that activate pDCs or mDCs via Toll-like receptors (TLR9 or TLR8). Activated DCs produce type I IFN, TNF-α, and cytokines (IL-6, IL-12, IL-23) which promote NETosis. Activated neutrophils produce LL-37 and nucleic acids, thereby self-propagating the inflammatory cycle.

Circulating auto-antibodies were also found in psoriasis patients. Human keratinocytes have Mas-related G-protein receptors. These receptors get continuously activated by LL-37 and β-defensins leading to the generation of cytokines and inflammation [32]. Studies show that PMNs respond quickly to RNA-LL-37 as compared to DNA-LL-37. Upon encountering RNA-LL-37 cytokines, chemokines, and NETs are released via TLR8 and TLR13. The NETs released by PMN also contain a high amount of RNA. Thus, PMNs and NET-derived RNA-LL-37 act as contributors to the self-propagating inflammatory cycle in psoriasis [67]. In psoriasis, LL-37 interacts with both self-DNA and self-RNA and translocates them to endosomal compartments of dendritic cells. Self-RNA-LL-37 triggers the activation of pDCs through TLR7 with the production of IFN-α, while self-DNA-LL-37 activates TLR9 with the release of IFN-α from pDCs. Self-RNA-LL-37 also activates TLR8 and in response releases inflammatory cytokines IL-6, IL-12, and IL-23 from mDCs (Figure 4). LL-37 acts as a stimulator to convert non-stimulatory self-RNA released from dying host cells to take part in innate immune activation. LL-37, when interacting with self-RNA forms, aggregates that protect the RNA against extracellular digestion by RNase [14,74]. In psoriasis, IL-17 is produced by innate immune cells and ETs found in human skin. Mast cells when treated with IL-23 and IL-1β in human skin readily form ETs with the release of IL-17. Mast cells and neutrophils have a significant role in psoriasis, as they release IL-17 through the formation of NETs [72] LL-37 which acts as an autoantigen in psoriasis and has the ability to directly initiate T cell activation. Apart from neutrophils and plasmacytoid dendritic cells (pDCs), other innate lymphocytes such as natural killer T (NKT) cells and natural killer (NK) cells that produce AMPs also have crucial roles in the development of psoriasis [61]. Human keratinocytes exhibit high levels of expression for Mas-related G protein-coupled receptors MRGPRX3 and MRGPRX4. The uncontrolled activation of these receptors by human defense peptides such as β-defensins and LL-37 can contribute to sensations of itch, pain, and other chronic inflammatory skin diseases [32]. In summary, AMPs and the cells that produce them play a significant part in the pathogenesis of psoriasis.

### 6.5. Vasculitis

Anti-neutrophil cytoplasmic autoantibody (ANCA)-associated vasculitis (AAV) refers to a group of autoimmune diseases that affect the entire body and are characterized by inflammation of small- to medium-sized blood vessels, causing necrotizing vasculitis [84]. Myeloperoxidase (MPO) and proteinase-3 (PR3) are proteins found in the lysosomes of monocytes and primary granules of neutrophils. When ANCA activates these cells, MPO and PR3 are released [85]. Zhang and colleagues were the first to discover that cathelicidin LL37 levels are elevated in AAV, particularly in patients with crescentic glomerulonephritis [86]. Cathelicidin LL37 is stored in an inactive form in the secondary granules of neutrophils and activated by PR3 (the target of PR3-ANCA) when released during inflammation [87]. When neutrophils and monocytes are activated by ANCA, they release MPO and PR3 along with other lysosome and granule proteins [85]. In addition, activated neutrophils also release cathelicidin LL37, which is found in neutrophil extracellular traps that have been linked to autoimmunity induction and inflammation mediation in AAV [88]. Therefore, cathelicidin LL37 can serve as valuable indicators in detecting active AAV, such as aggressive crescentic glomerulonephritis, making them possible prognostic markers to develop novel therapies [86].

### 6.6. Gout

Gout is a type of inflammatory arthritis accompanied by painful joints. It is caused when the body’s own inflammatory response is activated by the accumulation of monosodium urate (MSU) crystals in the joints. These crystals attract immune cells, known as leukocytes, and stimulate the production of NETs, which in turn lead to inflammation [89,90]. Auto-inflammatory disease such as gout has been associated with NETosis and aggregation of NETs. It is proposed that the clustering of NETs aids in the subsiding of inflammation caused by neutrophils by breaking down cytokines and chemokines and interfering with the recruitment and activation of neutrophils [91]. The presence of MSU crystals triggers an inflammatory response by activating the NALP3 inflammasome in macrophages that reside in the tissue. This, in turn, leads to the production of a significant amount of IL-1β. Consistent with previous research, the components NLRP3, ASC, Caspase-1, IL-1β, and IL-1R are all crucial factors in the inflammation and hypersensitivity that results from MSU crystal-induced joint inflammation [92,93]. The role of cathelicidin LL-37 on the activation of NLRP3 inflammasome is still not clear [94]. The complete understanding of the association between LL-37 and clinical markers, as well as pro-inflammatory mediators in individuals diagnosed with gout, remains uncertain.

## 7. AMPs as Signaling Molecules in Various ADs

AMPs as modulators of innate immune response function through their antimicrobial activity and also as signaling molecules via their chemotactic activity [25]. It has been well established through researched evidence that AMPs are dysregulated in various immune disorders, contributing to the pathophysiology of ADs [95]. Additionally, dysregulated production and impaired clearance of NETs stimulate dendritic cells through Toll-like receptors (TLR7 and TLR9) to produce interferons that enhance autoimmune responses by triggering antigen presentation and stimulating the production of autoantibodies by B-cells [96]. Upon receiving external stimuli, NETs get activated through protein kinase C (PKC)-Raf/MERK/ERK and NADPH oxidase (NOX) complex [97]. This is followed by the activation of myeloperoxidase (MPO), neutrophil elastase (NE), and protein arginine deiminase type 4 (PAD4) [98]. Together, these bring about the citrullination of histones and aid in the decondensation of chromatin. Additionally, ROS species promote NETosis through the release of chromatin outside these pores and the loss of nuclear membrane. This process finally gets completed upon cellular lysis and release of DNA, intracellular granules from extracellular traps, and citrullinated histones (citH3) [99]. Non-specific effects of these released enzymatic proteins contribute towards uncontrolled inflammatory responses in disease pathology. These negative effects are well understood through examples of disease pathology in ADs. Recruitment of neutrophils to psoriasis lesions leads to the formation of Munro’s microabscesses and spongiform pustules that produces pro-inflammatory cytokines such as interleukins (IL-17, IL-8, and IL-6) [100]. These inflammatory cytokines enhance NETosis and also the expression of LL-37 and defensins that further regulate NET formation [59]. SLE is further aggravated by autoantibodies generated against nucleic acids released by neutrophils undergoing NETosis [101]. In this context, these immune complexes serve as a reservoir of self-antigens that contribute to the enhancement of inflammatory processes and cause enhanced injury and inflammation [102].

NCFI-339 polymorphism is one of the factors known to alter the formation of NETs, and enhance serum interferon activity and antiphospholipid syndrome in SLE [103]. Patients with small vessel vasculitis (SVV), a condition of uncertain origin involving necrotizing inflammation of blood vessels, have demonstrated to harbor ANCAs that are predominantly produced due to proteins released through NETosis. This contributes to the activation of the complement system accompanied by endothelial damage. A-PR3 and α-MPO were found to be the ANCAs that lead to perpetuating feedback loops by activating NETosis during active disease conditions [104]. Gout is another autoimmune disorder that shares similarities with other ADs because of the involvement of neutrophil dysregulations. Proinflammatory substances derived from neutrophil granulocytes drive local acute immune responses against external pathogens but also act as endogenous inflammatory triggers [105]. During early periods of inflammation in gout, phosphatidylserine-positive neutrophil microvesicles cause the infiltration of neutrophils toward the peritoneum [106]. Furthering this, uric acid precipitation and crystallization leads to the activation of inflammasomes in local immune cells and enhanced recruitment of neutrophils. The clinical feature that is commonly observed in gout patients is primarily caused by the accumulation of activated neutrophils that are derived from NETs present in the synovial fluid. Autophagy and IL-1β are responsible for this accumulation process [107].

NETs can thus be thought of as efficient systems by virtue of their dual roles of trapping and exterminating extracellular pathogens and controlling their dissemination; the only problem lies in the dysregulation of their release, which can pave the way for various ADs. Hence, therapeutic management of NETs requires effective approaches that do not compromise the efficacy of NETs and at the same time, ensure that their dysregulated effects are avoided.

## 8. Role of AMPs in Medicine

With drug-resistant bacteria becoming more and more prominent, the broad-spectrum activity of AMPs with their multiple modes of action is proving to be a far better alternative. For instance, AMPs F(KW)2K and F(RI)2R with β-hairpin structure were synthesized with strong antibacterial properties, their therapeutic index (TI) being 70.7 and 115.9, respectively, for pulmonary bacterial infection. They do not have hemolytic toxicity in mice and prevent drug resistance when used combined with antibiotics [108]. The minimum inhibitory concentration (MIC) of the AMP WUL10 from the genus *Brevibacillus* is 1 μg/mL and the minimum bactericidal concentration (MBC) is 1–2 μg/mL against both methicillin-resistant *Staphylococcus aureus* (MRSA) and *S. aureus* TISTR 517. Upon creating a synthetic version, the MIC and MBC become 16 and 64 μg/mL, respectively, indicating that there is a natural synergistic effect due to other AMPs [109]. AMP SAAP-148 was designed as an improvement upon human cathelicidin LL-37 and its derivative OP-145 and tested against *Enterococcus hirae* and its membrane components. After a five-minute exposure, 0.4 μM of SAAP-148 and 3.2 μM of OP-145 were required to eradicate the bacteria by depolarization and permeabilization of membrane leading to cell shrinkage and other abnormalities, thereby preventing the thermotropic phase transition of the bilayer [110]. Over 30 AMP-based drugs have reached clinical trials. It is seen that the use of mainstream antibiotics together with AMP drugs has a compounded microbicidal effect [111]. For patients who have undergone abdominal surgery, postoperative adhesion due to tissue healing and trauma has proven to be a major challenge. For this, a hydrogel J-1-ADP was created with the natural jelleine-1 (J-1) AMP, which self-assembles in platelet-activating factor adenosine diphosphate (ADP) sodium solution. This has antibacterial and antifungal properties and is also biocompatible and degradable. The hydrogel also promotes wound healing through hemostatic activity in mouse liver hemorrhage models, as seen by platelet activation, platelet adhesion assays, plasma coagulation, and whole blood coagulation. Another model of rat sidewall defect-cecum abrasion also exhibited an anti-adhesion effect [112]. With regard to medical implants, AMPs have been used for osseointegration, where the implant is fused to the bone. Silver nanoparticles were used for surface complexation of AMPs with osteogenic fragments, carried by collagen structure bionic silk fibroin. This gave it 99% antibacterial properties against *S. aureus* for 21 days through ROS production and cell membrane destruction [113].

Given the high prevalence of nosocomial infections, AMPs are suitable for the surface functionalization of medical devices to prevent bacterial biofilm formation. Polydimethylsiloxane polymer is loaded with the AMP, human apolipoprotein B [r(P)ApoBLPro], giving it anti-adhesive properties [114] with MBC of 80 μM against *S. aureus* ATCC 29213 and 10 μM against *Escherichia coli* ATCC 25922. Human dermal fibroblasts (HDFs) and Murine BALB/c-3T3 fibroblasts were subjected to MTT assay to determine if the system had an incompatibility with eukaryotic cells. At the highest concentration, it showed 20% cell death, indicating slight toxicity. AMPs are, however, susceptible to proteolytic cleavage, hindering their use clinically. To remediate this, nanomaterial networks are used as scaffolds to protect them from enzymatic action. Moldable, colloidal nano-networks of chitosan and dextran sulfate were fabricated to deliver AMP PA-13 locally. This was electrostatically encapsulated in biodegradable materials and was capable of destroying *Pseudomonas aeruginosa* in vitro and ex vivo on a porcine skin model [115].

## 9. Conclusions

With the emergence of multi-drug-resistant microbes, there has been an urgency in finding alternatives to antibiotics. AMPs could be the answer to this problem. Apart from their antimicrobial activity, they play a crucial role in the regulation of immune responses. AMPs such as defensin and cathelicidin when bound with self-DNA or self-RNA act as an autoantigen, thereby invoking the auto-inflammatory pathways, thus favoring the development of ADs. The intriguing role of AMPs in the development of ADs renders them captivating targets for further investigation. The pursuit of drugs and biomarkers for the treatment of ADs holds promise, but caution is warranted as the dysregulation of AMPs can potentially contribute to the development of multiple ADs. To summarize, in-depth research and a detailed understanding of their role in the progress of ADs are crucial steps prior to the progress of novel therapeutic approaches involving AMPs. The rapid progress in the field of biopolymer research and nanotechnology is indeed opening newer options for the use of AMPs with targeted and extended-release options.

## Figures and Tables

**Table 1:** List of major antimicrobial peptides (AMPs) playing a role in innate immunity.

Peptides	Amino Acid Composition	Conserved Length/M.W.	Source	Activity
Human neutrophil antimicrobial peptide (HNP-1, HNP-2, HNP-3) or Defensins	Rich in cysteine, arginine, and aromatic residues	29–30 residues in length, 11 conserved residues (including 6 cysteinyl residues)	Azurophil granules of human PMN	Antibacterial, antiviral, antifungal [33,34]
Rabbit peptide (NP-1, NP-2, NP-3a, NP-3b, NP-4, NP-5)	Rich in cysteine, arginine, and absence of aromatic amino acids	32–34 residues in length, 11 conserved residues (including 6 cysteinyl residues)	Rabbit PMN	Antibacterial, antiviral, antifungal [35]
Rabbit macrophage cationic peptides MCP-1, MCP-2	Rich in cysteine, arginine	30–33 residues long with three intramolecular disulfide bond	Rabbit macrophage	Antibacterial [35]
Mytilin	Cysteine-rich peptide	34 residue (3.7 kDa)	Blood of mussels (*Mytilus edulis*)	Antibacterial, antifungal [36]
Human β-Defensins (HBD 1–4)	Cysteine, arginine, lysine-rich peptide	38–42 residues	Bovine tracheal epithelium, Keratinocytes	Antibacterial [37]
Human α-Defensins (HD-5 and HD-6)	Cysteine and arginine-rich peptide	29–35 residues	Small intestine, colon, and female genital epithelium cells	Antibacterial, antifungal [38]
Indolicidine	Tryptophan and proline-rich peptide	13 residues	Bovine neutrophils	Antibacterial [37,39]
Bactenecins (Bac7 and Bac5)	Proline and arginine-rich peptides	40–60 residues	Bovine neutrophils	Antibacterial, antiviral [37,40]
Cathelicidin antimicrobial peptide (CAMP), human LL-37/hCAP18, and mouse CRAMP (cathelicidin-related antimicrobial peptide)	Proline and arginine-rich peptide	37 residues	Leukocytes, neutrophils, pancreatic β-cells various body cells, tissues, and body fluids	Antibacterial, antifungal antiviral activity, regulating inflammatory response, acts as chemo-attractant, neutralizing LPS [3,41]
Porcine NK-Lysine (PNKL)	Cysteine and lysine, leucine-rich peptide	145 amino acid (33 kDa)	Small intestine	Intestinal inflammatory response [42]
Lactoferrin	Iron-binding glycoprotein	80 kDa	Mucosal layer	Antiviral activity against SARS-CoV [43]

## Data Availability

All the data and material generated and analyzed in this study have been included in this manuscript. The data presented in this study are available on request from the corresponding authors.

## Abbreviations

AMP: antimicrobial peptide; NET: neutrophil extracellular trap; AD: autoimmune disorder; DNA: deoxyribonucleic acid; RNA: ribonucleic acid; NK: natural Killer; LPS: lipopolysaccharide; ROS: reactive oxygen species; NADPH: nicotinamide adenine dinucleotide phosphate; pDC: plasmacytoid dendritic cell; NK: natural killer; Tc-cells: cytotoxic T-cells; CAMP: cathelicidine antimicrobial peptide; TLR: Toll-like receptor; SEM: scanning electron microscopy; PMA: Phorbol-12- myristate-13-acetate; PKC: Protein kinase C; HNP: human neutrophil peptide; HBD: human β-defensins; TNF: tumor necrosis factor; hCAP: human cationic antimicrobial protein; PMN: polymorphonuclear neutrophil; MCP: monocyte chemoattractant protein; PNKL: porcine NK-lysine; WBC: white blood corpuscle; IDR: innate defense regulators; FPRL1: formyl peptide receptor-like 1; iDC: immature dendritic cells; GPCR: G-protein coupled receptor; CCR6: C-C chemokine receptor type 6; CXCR4: C-X-C chemokine receptor type 4; HIV: human immunodeficiency virus; IL: interleukin; IFN: interferon; SLE: systemic lupus erythematosus; cAMP: cyclic adenosine monophosphate; MC: mast cell; MIP: macrophage inflammatory protein; IP: interferon-gamma-inducible protein; CD: cluster of differentiation; APC: antigen presenting cell; CRISPR: clustered regularly interspaced short palindromic repeats; RA: rheumatoid arthritis; CRAMP: cathelicidin-related antimicrobial peptide; SARS-CoV2: severe acute respiratory syndrome coronavirus 2; MPO: myeloperoxidase; FDA: Food and Drug Administration; CLP: cecal ligation and puncture; FPRL1: formyl peptide receptor-like 1; GAS: group A streptococci; mDCs: myeloid dendritic cells; TIDM: type I diabetes mellitus; NOD: non-obese diabetics; MRGPRX: Mas-related G protein-coupled receptors; ANCAs: anti-neutrophil cytoplasmic antibodies; AAV: associated vasculitis; PR3: proteinase-3; MSU: monosodium urate; MEK/ERK: mitogen-activated ERK kinase; NOX: NADPH oxidase; NE: neutrophil elastase; PAD4: protein arginine deiminase type 4; citH3: citrullinated histones; SVV: small vessel vasculitis; MBC: minimum bactericidal concentration; MRSA: methicillin-resistant staphylococcus aureus; ADP: adenosine diphosphate; HDFs: human dermal fibroblasts; MTT: 3-(4,5-dimethylthiazol-2-yl)-2,5-diphenyl-2H-tetrazolium bromide.
